# Metabonomics study of the effects of single copy mutant KRAS in the presence or absence of WT allele using human HCT116 isogenic cell lines

**DOI:** 10.1007/s11306-021-01852-w

**Published:** 2021-11-25

**Authors:** Dorna Varshavi, Dorsa Varshavi, Nicola McCarthy, Kirill Veselkov, Hector C. Keun, Jeremy R. Everett

**Affiliations:** 1 Medway Metabonomics Research Group, University of Greenwich, Chatham Maritime, ME4 4TB Kent UK; 2 Present Address: Department of Biological Sciences, University of Alberta, 116 Street & 85 Ave, Edmonton, AB T6G 2R3 Canada; 3 Horizon Discovery Ltd., Cambridge Research Park, 8100 Beach Dr, Waterbeach, Cambridge, CB25 9TL UK; 4 Present Address: Milner Therapeutics Institute, Jeffrey Cheah Biomedical Centre, University of Cambridge, Puddicombe Way, Cambridge, CB2 0AW UK; 5 Department of Surgery and Cancer, Faculty of Medicine, Imperial College, London, SW7 2AZ UK; 6 Department of Surgery and Cancer, Imperial College London, Hammersmith Hospital Campus, London, W12 ONN UK

**Keywords:** KRAS, Mutations, HCT116, Cells, Colorectal cancer, Metabonomics, Metabolomics, Metabolic profiling, NMR

## Abstract

**Introduction:**

*KRAS* was one of the earliest human oncogenes to be described and is one of the most commonly mutated genes in different human cancers, including colorectal cancer. Despite *KRAS* mutants being known driver mutations, *KRAS* has proved difficult to target therapeutically, necessitating a comprehensive understanding of the molecular mechanisms underlying *KRAS*-driven cellular transformation.

**Objectives:**

To investigate the metabolic signatures associated with single copy mutant *KRAS* in isogenic human colorectal cancer cells and to determine what metabolic pathways are affected.

**Methods:**

Using NMR-based metabonomics, we compared wildtype (WT)-*KRAS* and mutant *KRAS* effects on cancer cell metabolism using metabolic profiling of the parental *KRAS* ^G13D/+^ HCT116 cell line and its isogenic, derivative cell lines *KRAS* ^+/–^ and *KRAS* ^G13D/–^.

**Results:**

Mutation in the *KRAS* oncogene leads to a general metabolic remodelling to sustain growth and counter stress, including alterations in the metabolism of amino acids and enhanced glutathione biosynthesis. Additionally, we show that *KRAS*^*G13D/*+^ and *KRAS*^*G13D/−*^ cells have a distinct metabolic profile characterized by dysregulation of TCA cycle, up-regulation of glycolysis and glutathione metabolism pathway as well as increased glutamine uptake and acetate utilization.

**Conclusions:**

Our study showed the effect of a single point mutation in one *KRAS* allele and *KRAS* allele loss in an isogenic genetic background, hence avoiding confounding genetic factors. Metabolic differences among different *KRAS* mutations might play a role in their different responses to anticancer treatments and hence could be exploited as novel metabolic vulnerabilities to develop more effective therapies against oncogenic *KRAS.*

**Graphical abstract:**

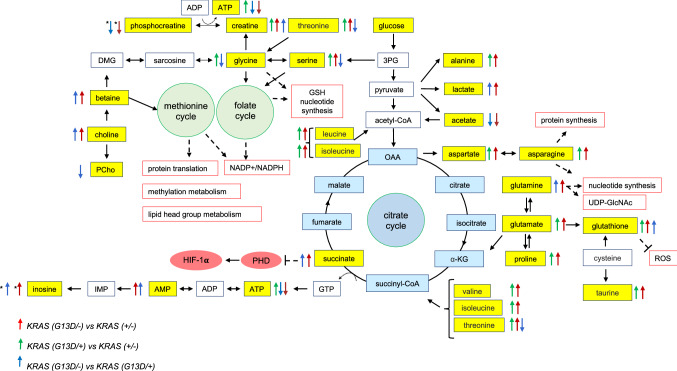

**Supplementary Information:**

The online version contains supplementary material available at 10.1007/s11306-021-01852-w.

## Introduction

Kirsten Rat Sarcoma Viral Oncogene Homolog *(Kras or kras2)* was one of the earliest human oncogenes to be described and is one of the most commonly mutated genes in different human cancers, including colorectal cancer (Dinu et al., 2014; Tsuchida et al., 2016). Activation of Ras is associated with a wide range of biological responses, such as cell growth, proliferation, survival, differentiation and tissue morphogenesis, depending on the cell type and stimuli (Vartanian et al., 2013). *KRAS* mutations are associated with poor prognosis and resistance to targeted therapies, demonstrating the clinical importance of this gene (Arrington et al., 2012).

In addition to the well-documented pro-tumorigenic role of mutant Ras alleles, there is some evidence suggesting that the wild type copy of Ras can display tumour-suppressor effects. Zhang et al*.* showed that following exposure to carcinogenic chemicals, mice harbouring only one copy of wild type *Kras2* produced larger lung tumors than mice harbouring both wild type alleles (Zhongqiu Zhang et al., 2001). To investigate the effect of a single copy mutant *KRAS*, Vartanian et al. analysed the signalling and biological properties of a panel of isogenic cancer cell lines (DLD1, HCT116, and Hec1A) (Vartanian et al., 2013). They demonstrated that a single copy of mutant *KRAS* results in only modest activation of downstream signalling pathways involving extracellular signal-regulated kinase (ERK) and AKT. One copy of mutant *KRAS* had a small effect on cell proliferation and cell migration, but a greater effect on cell transformation, as evaluated by growth in soft agar. Interestingly, elimination of the wild type *KRAS* allele led to increased growth in soft agar, indicating tumor-suppressive characteristics of the wild type *KRAS* allele under these conditions (Vartanian et al., 2013).

Like many oncogenes, mutant *KRAS* impacts cellular metabolism and these effects might underlie some of the biological effects of wild type and mutant *KRAS* outlined above.

Metabonomics (Everett et al., 2019; Lindon et al., 2000) is a novel field in systems biology that produces disease-relevant molecular information downstream of the genome and proteome and has shown early promise in cancer research. We recently demonstrated the power of metabonomics to define the specific profiles of human SW48 colorectal cancer cells carrying different *KRAS* mutations in codons 12, 13, 61 and 146 compared with their wild-type counterpart (Varshavi et al., 2020).

In this study, we applied NMR-based metabonomics to obtain an understanding of metabolic dysregulation driven by common *KRAS* mutations and also studied the effects of single copy mutant *KRAS* on the cellular metabolic profile. Specifically, we compared the metabolic profiles of parental HCT116 *KRAS *^*G13D/*+^ (a cell line isolated from a patient with colorectal cancer in which glycine (G) 13 is mutated to aspartate (D)) and its isogenic derivative cell lines: *KRAS* ^+*/–*^ and *KRAS *^*G13D/–*^. In the cell line *KRAS* ^+*/–*^*,* the mutated *KRAS* allele is deleted and the WT allele remains, whereas in *KRAS *^*G13D/–*^ the WT *KRAS* allele is deleted and the G13D mutant allele remains. These isogenic cell lines share the same genetic background, differing only by the mutational status of the *KRAS* gene and therefore allowed study of the metabolic functions of normal or mutant KRAS proteins and in particular, allowed us to determine the effects of the loss of an allele of the cell line metabolic profile, something that had not been possible in our previous studies (Varshavi et al., 2020).

## Experimental

### Cell culture and harvesting method

HCT116 colorectal carcinoma isogenic cell lines were obtained from Horizon Discovery Ltd (Cambridge, UK) and maintained at 37 °C with 5% CO_2_. Cell lines were generated using the Recombinant Adeno-Associated Virus (rAAV) based gene editing platform and were all validated by genotyping—gDNA and cDNA. The study was conducted in vitro with the cells transiently growing as spheroids in low adherence flasks, cultured and harvested as recently described using five biological replicates (Varshavi et al., 2020).

### Metabolite extraction of HCT116 cell lines

Intracellular metabolites were extracted as previously described with some modifications (Dettmer et al., 2011). 800 μl of chilled MeOH/H_2_O (− 20 °C, 80:20(v/v)) was added to the frozen cell pellet, homogenized for 1 min using Pellet pestles (Sigma-Aldrich, UK). The sample was centrifuged at 4 °C and 7000×*g* for 5 min and the supernatant was collected into a new Eppendorf tube. The pellet was re-extracted three times with 400 μl cold methanol/water (− 20 °C, 80:20 (v/v)) to produce 2 ml of extract. The supernatants were combined, dried under a gentle flow of N_2_ gas and stored at -80° C until dissolution for NMR analysis.

### Cell extract sample preparation and NMR acquisition

Samples were prepared, acquired, processed and analysed as described previously (Benjamini, 2010; Varshavi et al., 2018, 2020; Veselkov et al., 2014). Two-dimensional NMR experiments were carried out for selected samples to aid/confirm the identities of the metabolites (Dona et al., 2016). Details for HCT116 KRAS ^G13D/−^ are in Supplementary Table 1. Metabolites were identified at MSI level 2 (Sumner et al., 2007) and as previously described and using published methods (Dona et al., 2016; Everett, 2015; Varshavi et al., 2020) (Table 1, Supplementary Table 2).

**Table 1:** Summary of the most significant metabolites differentiating between the genotypes *KRAS* ^G13D/+^ vs *KRAS*^±^, *KRAS* ^G13D/−^ vs *KRAS*^±^ and *KRAS* ^G13D/−^ vs *KRAS* ^G13D/+^ (*p* value adjusted for FDR of 0.1)

Metabolite	Chemical shifts in ppm	*KRAS*^*G13D/*+^ *vs KRAS*^±^	*KRAS* ^G13D/−^ vs *KRAS*^±^	*KRAS* ^G13D/−^ vs *KRAS* ^G13D/+^
Isoleucine	0.943 (t), 1.01 (d), 3.675 (d)	↑	↑	–
Valine	0.996 (d), 1.046 (d), 3.615 (d)	↑	↑	–
Leucine	0.961 (d), 0.972 (d), 1.691 (m), 1.720 (m), 1.748 (m), 3.737(dd)	↑	↑	–
Lactate	1.331 (d), 4.113 (q)	–	↑	↑
Threonine	1.335 (d), 3.591 (d)	↑	↑	↓
Alanine	1.48 (d), 3.787 (q)	↑	↑	–
Acetate	1.919 (s)	–	↓	↓
Proline	2.011 (m), 2.073 (m), 2.359 (m), 4.14 (dd)	↑	↑	–
Glutamate	2.059 (m), 2.140 (m), 2.355 (m), 3.761 (dd)	↑	↑	–
Glutamine	2.145 (m), 2.46 (m), 3.783 (t)	–	↑	↑
Glutathione	2.171 (m), 2.560 (m), 2.935 (dd), 2.980 (dd), 3.782 (m), 4.572 (dd)	↑	↑	↑
Succinate	2.406 (s)	–	↑	↑
Aspartate	2.684 (dd), 2.816 (dd), 3.902 (dd)	↑	↑	–
Asparagine	2.85 (dd), 2.88 (dd)	↑	↑	–
Creatine	3.041 (s)	↑	↑	↑
Creatine phosphate	3.045 (s), 3.95 (s)	–	↓	↓#
Choline	3.207 (s)	–	↑	↑
Phosphocholine	3.224 (s), 3.597 (s)	–	–	↓
Betaine	3.269 (s)	–	↑	↑
Taurine	3.27 (t), 3.42 (t)	↑	↑	–
Myo-inositol	3.284 (t), 3.54 (dd), 3.62 (dd), 4.067 (t)	↓	–	↑
Glycine	3.562 (s)	↑	–	↓
Serine	3.848 (dd), 3.950 (dd), 3.995 (dd)	↑	↑	↓
Inosine	3.845 (dd), 3.915 (dd), 4.282 (m), 4.442 (dd), 4.787 (t), 6.107 (dd), 8. 241 (s), 8.348 (s)	–	↑♦	
ATP	8.54 (s), 8.27 (s)	↑	↓	↓
AMP	8.61 (s), 8.27 (s)	–	↑	↑

Footnotes: # Absent in *KRAS*^*G13D/−*^ ♦ Absent in *KRAS*^±^  Absent in *KRAS*^*G13D/*+^

### Multivariate analysis of HCT116 cell line extracts

PCA was conducted on all samples to establish any obvious outliers in the data and to determine any between group differences. In order to maximize the separation between classes, NMR data were also subject to a supervised dimension-reduction technique, maximum margin criterion (MMC, Supplementary Fig. 1) (Veselkov et al., 2014). Leave-one-out cross-validation with the quadratic as a classifier was applied to check the validity of the MMC model (Supplementary Fig. 2). To determine metabolites responsible for the class separation, one-way ANOVA was applied with a false discovery rate (FDR) of 0.1 i.e. 10%, in order to account for multiple hypothesis testing.

## Results

### Metabonomics analysis of HCT116 cell line extracts expressing WT or G13D mutant *KRAS*

**Fig. 1:**
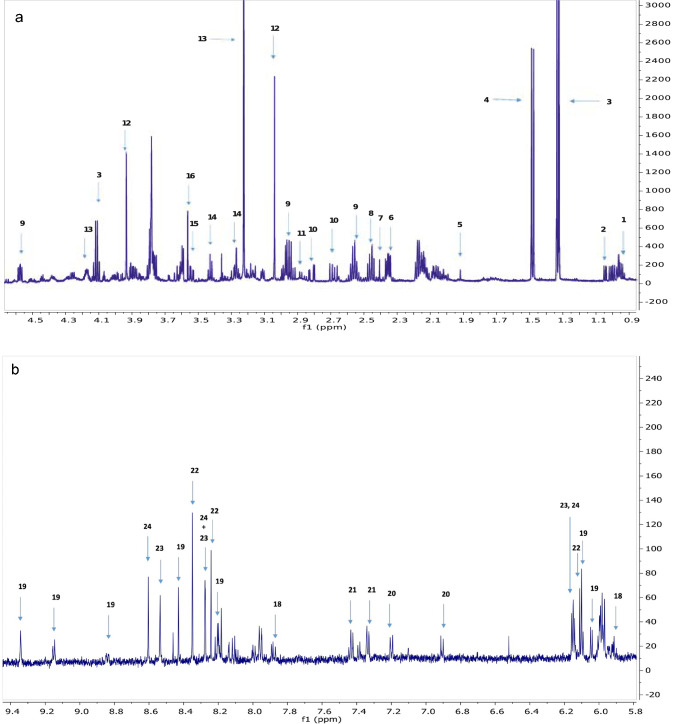
**a** The aliphatic region of the 600 MHz ^1^H NMR spectrum from an HCT116 *KRAS* ^G13D/−^ extract. Numbers indicate signals corresponding to individual metabolites. 1. leucine; 2. valine; 3. lactate and threonine; 4. alanine; 5. acetate; 6. glutamate; 7. succinate; 8. glutamine; 9. glutathione; 10. aspartate; 11. asparagine; 12. creatine; 13. O-phosphocholine; 14. taurine; 15. myo-inositol; 16. glycine. **b** The aromatic region of the 600 MHz ^1^H NMR spectrum from an HCT116 *KRAS* ^G13D/−^ extract. Numbers indicate signals corresponding to individual metabolites. 18. uridine; 19. NAD; 20. tyrosine; 21. phenylalanine; 22. inosine; 23. ATP; 24. AMP

**Fig. 2:**
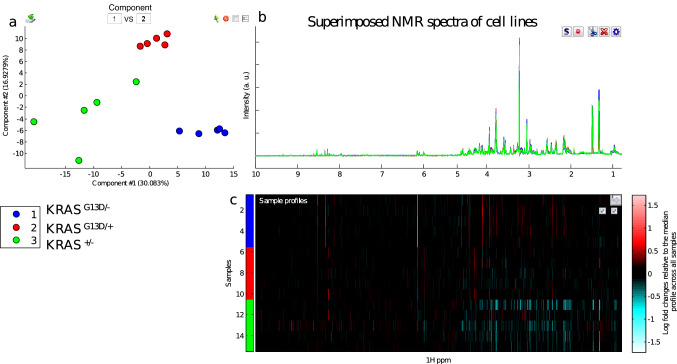
**a** PCA score plot of the 600 MHz ^1^H NMR spectra of extracts of HCT116 cells, **b** superimposed NMR spectra of cells, with the same colour coding as in the PCA plot; **c** ‘heat map display of the 600 MHz ^1^H NMR spectra from 0.8 to 10.0 ppm of *KRAS *^G13D/−^ (top 5 strips), *KRAS* ^G13D/+^middle 5 strips) and *KRAS* ^±^ (bottom 5 strips). Red and blue elements in the spectra indicate NMR signals that are more intense, or less intense, respectively, than the median signal intensity for all the samples

A representative ^1^H NMR spectrum from an HCT116 *KRAS* ^G13D/−^ extract is shown in Fig. 1 (a, b). PCA of the three genotypes showed high degrees of reproducibility among the biological replicates for each group. Moreover, PCA demonstrated a clear discrimination between all three genotypes: *KRAS* ^G13D/+^, *KRAS* ^±^ and *KRAS* ^G13D/−^ as shown in Fig. 2. Supervised maximum margin criterion (MMC) analysis gave correct classification rates of 100% for the models constructed between all classes.

**Fig. 3:**
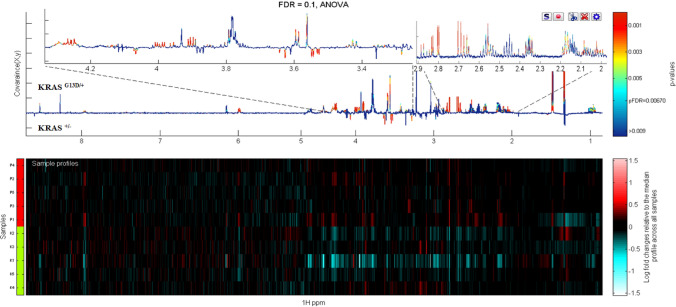
At bottom a ‘heat map display of the 600 MHz ^1^H NMR spectra of *KRAS* ^G13D/+^ (top 5 strips) vs the corresponding spectra of *KRAS*^±^ (bottom 5 strips). Red and blue elements in the spectra indicate NMR signals that are more intense, or less intense, respectively, than the median signal intensity for all the samples. At top, the corresponding ANOVA plot, showing positive peaks for those metabolite signals that are more intense in *KRAS* ^G13D/+^, and negative peaks for those metabolite signals that are less intense. The signals are colour coded by the p value adjusted for FDR of 0.1

**Fig. 4:**
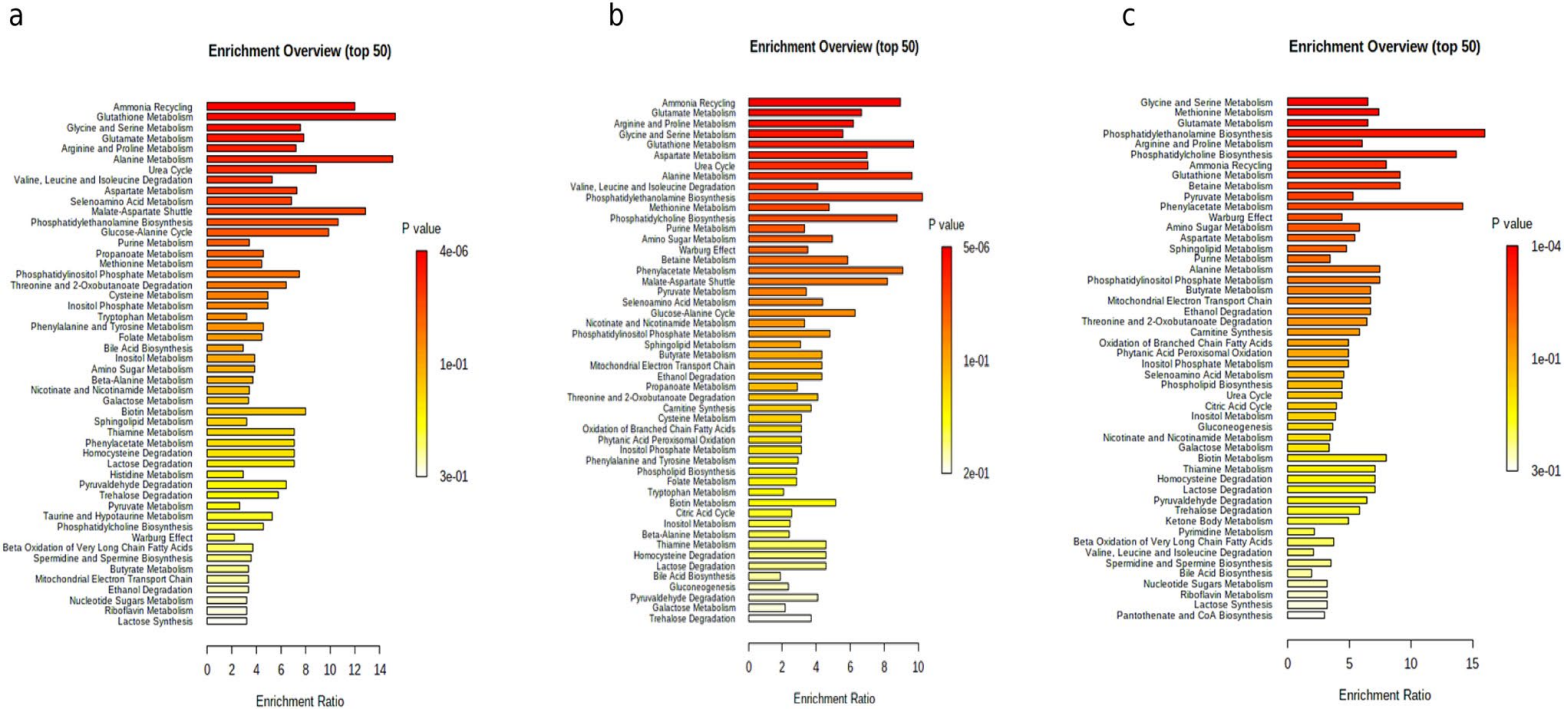
Metabolic pathway analyses related to the metabolites that were discriminating between **a** KRAS ^G13D/+^ and KRAS^±^, **b** KRAS ^G13D/−^ and KRAS^±^ and **c** KRAS ^G13D/+^ and KRAS ^G13D/−^. The horizontal bars summarize the main metabolite sets identified in this analysis; the bars are coloured based on their p values and the length is based on the fold enrichment

Figure 3 and Supplementary Figs. 3 and 4 show representative ANOVAs built between genotypes (*KRAS* ^G13D/+^ vs *KRAS* ^±^), (*KRAS* ^G13D/−^ vs *KRAS* ^±^) and (*KRAS* ^G13D/+^ vs *KRAS* ^G13D/−^) respectively. Table 1 and Supplementary Table 3 provide a list of statistically significant metabolites and their relative fold-changes between genotypes. To further understand the biological significance of the metabolite changes in the KRAS mutant clones, we used enrichment analysis (EA) tools in MetaboAnalyst to link metabolites to metabolic pathways (Fig. 4). In both KRAS ^G13D/−^ and KRAS ^G13D/+^ cells, ammonia recycling, glutathione metabolism, glycine and serine metabolism pathways and glutamate metabolism were over-represented.

*KRAS* ^G13D/+^ and *KRAS* ^G13D/−^ cells showed elevated levels of valine, isoleucine, leucine, threonine, alanine, proline, glutamate, glutathione, aspartate, asparagine, creatine, taurine and serine relative to HCT116 cell lines harbouring only one WT *KRAS* allele (*KRAS* ^±^).

Compared to *KRAS* ^±^ cells, *KRAS* ^G13D/−^ cells also exhibited higher levels of lactate, glutamine, succinate, choline, betaine, inosine and adenosine 5′-monophosphate (AMP) and lower levels of acetate, creatine phosphate and adenosine 5′-triphosphate ATP while *KRAS* ^G13D/+^ cells displayed increased levels of glycine and ATP and decreased levels of myo-inositol.

*KRAS* ^G13D/−^ cells showed higher levels of lactate, succinate, choline, betaine, glutamine, glutathione, creatine and inosine, and lower levels of serine, threonine, glycine, acetate, creatine phosphate, phosphocholine, myo-inositol and ATP relative to *KRAS* ^G13D/+^ cells.

## Discussion

Altered metabolism has been recently recognized as one of the hallmarks of cancer cells, allowing metabonomics to be widely used as a powerful tool to identify diagnostic cancer biomarkers and new therapeutic targets (Spratlin et al., 2009; Varshavi et al., 2020; Vermeersch & Styczynski, 2013). Oncogenic *KRAS* is one of the genes that is frequently mutated in colorectal cancer, and mutant *KRAS* is associated with poor prognosis and resistance to therapeutics (Mayers et al., 2016). About 80% of *KRAS* mutations are heterozygous (Hartman et al., 2012). However, in many types of cancer the remaining wild-type *KRAS* gene can be lost during tumour progression. Therefore, wild-type *KRAS* might serve as a tumour-suppressor gene in the presence of mutant *KRAS* (Luo et al., 2014; To et al., 2013; Zhang et al., 2001; Zhongqiu Hartman et al., 2012). Mutant *KRAS* is a known driver mutation for cancer development and progression, making KRAS mutant proteins good candidates for therapeutic development. Yet, until recently, KRAS mutants have proved difficult to target with small molecules, fuelling interest in targets upstream and downstream of *KRAS* for drug discovery. Understanding the metabolic consequences of WT *KRAS* allele loss might provide additional information in identifying key vulnerabilities in tumours with mutant KRAS.

In the current study, we applied an exploratory, untargeted, NMR-based metabonomics approach to obtain an understanding of metabolic dysregulation driven by a common human *KRAS* oncogenic mutation, G13D. The use of isogenic cell lines in this study also afforded us window into the effects of single copy mutant *KRAS* on metabolic alteration.

To this end, we compared the metabolic profiles of the parental *KRAS* ^G13D/+^ (parental line isolated from a colorectal cancer patient in which glycine (G) 13 is mutated to aspartate (D)) and its isogenic derivative cell lines *KRAS* ^+/–^ and *KRAS* ^G13D/–^ in human colon cancer cells (HCT116). In the cell line *KRAS* ^+/–^, the mutated *KRAS* allele is deleted and the WT one remains, whereas in *KRAS* ^G13D/–^ the WT *KRAS* allele is deleted and the G13D mutated one remains.

**Fig. 5:**
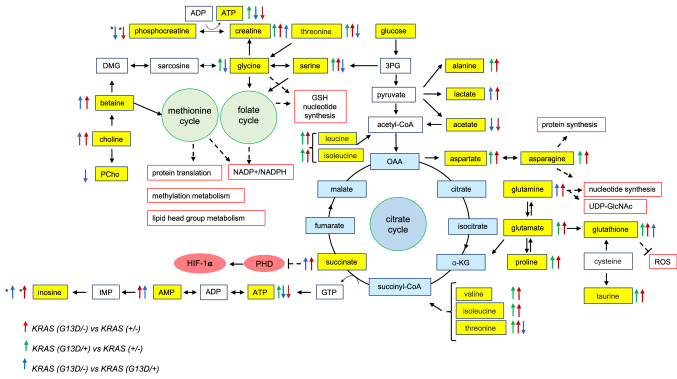
An overview of metabolic pathways altered in HCT116 cell line expressing the WT or the G13D mutant *KRAS*. Yellow boxes represent deregulated metabolites. The direction of the arrows shows the regulation direction of metabolites; the red arrows represent metabolites in *KRAS* ^G13D/−^ compared to *KRAS* ^±^; the green arrows represent metabolites in *KRAS* ^G13D/+^ compared to *KRAS* ^±^, the blue arrows represent metabolites in *KRAS* ^G13D/−^ compared to *KRAS* ^G13D/+^.  indicates that the metabolite is only present or only absent in *KRAS* ^G13D/−^ compared to *KRAS* ^G13D/+^ respectively.  indicates that the metabolite is only present and absent in *KRAS* ^G13D/−^ compared to *KRAS* ^±^ respectively: 3PG (3-phosphoglycerate), AMP (Adenosine monophosphate), ADP (Adenosine diphosphate), ATP (Adenosine triphosphate), α- KG (α-ketoglutarate), DMG (Dimethylglycine), GSH (Glutathione), GTP (Guanosine-5'-triphosphate), HIF-1α (Hypoxia-inducible factor 1-alpha), NADP (Nicotinamide adenine dinucleotide phosphate), NADPH (Nicotinamide adenine dinucleotide phosphate), PCho (Phosphocholine), PHD (Prolyl-hydroxylase), ROS (Reactive Oxygen Species), UDP-GlcNAc (Uridine diphosphate *N*-acetylglucosamine)

PCA analysis (Fig. 2) showed a clear discrimination not only between mutant-containing (*KRAS* ^G13D/+^, *KRAS* ^G13D/–^) and wild-type-containing (*KRAS* ^+/–^) *KRAS,* but also between *KRAS* ^G13D/+^ and *KRAS* ^G13D/–^. Interestingly, parental cell lines (*KRAS* ^G13D/+^) clustered more closely to mutant *KRAS*-deleted lines (*KRAS* ^+/–^) than to WT *KRAS*-deleted lines (*KRAS* ^G13D/–^), indicating that in each isogenic cell line the WT *KRAS* allele has a stronger effect on the overall metabolic profile than the mutant allele. The main alterations associated with mutant *KRAS* involved changes in multiple metabolic pathways such as glycolysis, TCA, and most significantly amino acid pathways (Fig. 5).

The amino acids most commonly consumed at a high rate by cancer cells are glutamine and serine (Tsun & Possemato, 2015). The serine levels in HCT116 *KRAS* ^G13D/+^ or *KRAS* ^G13D/–^ cells were significantly higher compared with isogenic HCT116 cells containing only a wild-type *KRAS* allele (*KRAS* ^+/–^).

Serine is a non-essential amino acid that contributes to a range of metabolic processes, which are vital for the growth and survival of cancer cells, including protein, lipid, nucleotide and glutathione biosynthesis, as well as one-carbon metabolism (Amelio et al., 2014; Yang & Vousden, 2016). Serine can be either taken up from the medium by the transformed cell or be produced from the glycolytic intermediate 3-phosphoglycerate (3PG). The first step in the serine synthesis pathway (SSP) involves the conversion of 3-phosphoglycerate to 3-phosphohydroxypyruvate (3PHP) by the NAD^+^-dependent enzyme 3-phosphoglycerate dehydrogenase (PHGDH). 3-Hydroxypyruvate can be then transaminated to phosphoserine by the enzyme phosphoserine aminotransferase (PSAT1), using glutamate as amino donor (Amelio et al., 2014; Yang & Vousden, 2016). Serine is subsequently produced from phosphoserine by phosphoserine phosphatase (PSPH). PHGDH, the initial enzyme in the SSP, is reportedly upregulated in breast cancers and melanomas (Labuschagne et al., 2014; Possemato et al., 2011). However, many cancer cells are largely dependent on uptake of exogenous serine, as de novo serine synthesis alone cannot support their proliferation and survival (Jain et al., 2012; Maddocks et al., 2013). The enhanced concentrations of serine in *KRAS* ^G13D/–^ and *KRAS* ^G13D/+^ cells relative to *KRAS* ^+/–^ cells could, therefore, be due to an increase in serine uptake from the medium and/or biosynthesis. Interestingly, lower levels of serine were observed in *KRAS* ^G13D/–^ versus *KRAS* ^G13D/+^ cells. This could be associated with either increased consumption or decreased uptake/biosynthesis. The latter is more likely, as serine is involved in adenine synthesis, and its limited availability can result in a decreased concentration of ATP in the cell. This is consistent with our observation, showing lower levels of ATP in *KRAS* ^G13D/–^ cells versus *KRAS* ^G13D/+^ and *KRAS* ^+/–^ cells. This hypothesis could be tested further by comparison of the serine concentration in the culture media across the cell lines.

Concentrations of glutamine in *KRAS* ^G13D/+^ and *KRAS* ^+/–^ cells were comparable but increased in *KRAS* ^G13D/–^ cells relative to *KRAS* ^+/–^ and *KRAS* ^G13D/+^ cells. The increased levels of glutamine in *KRAS* ^G13D/–^ cells could indicate that the WT *KRAS*-deleted isogenic cell lines are more dependent on glutamine and hence they take it up at a high rate.

Glutamine is a “conditionally essential” amino acid that is synthesized sufficiently under normal circumstances but becomes essential during proliferation. *KRAS*-mutant cells can be particularly dependent on glutamine as a carbon source to fuel the TCA cycle and as a nitrogen source for nucleotide, hexosamine and nonessential amino acid (NEAA) biosynthesis (Toda et al., 2016). Glutamine is transported into the cell through one of many transporters, such as the neutral amino acid transporter (ASCT2) (Scopelliti et al., 2018). Once imported into the cells, glutamine is converted to glutamate by glutaminase (GLS) in the mitochondria (Chendong Yang et al., 2014). In this study, higher intracellular concentrations of glutamate were observed in *KRAS* mutant cells (*KRAS* ^G13D/+^, *KRAS* ^G13D/–^) relative to wild-type-containing cells (*KRAS* ^+/–^), indicating increased glutaminolysis.

Glutamate can subsequently be converted to α-ketoglutarate by glutamate dehydrogenase 1 (GLUD1) where it can fuel the TCA cycle or be converted to nonessential amino acids such as aspartate and alanine by transaminases, including glutamate–oxaloacetate transaminase (GOT) and glutamate–pyruvate transaminase (GPT), respectively. Glutamate can also contribute to the synthesis of proline and glutathione (Liu et al., 2012).

In the current study, *KRAS* ^G13D/+^ and *KRAS* ^G13D/–^ cells showed increased levels of metabolites associated with the glutamate synthase cycle including alanine, aspartate, asparagine, proline and glutathione when compared to *KRAS* ^+/–^ cells.

Elevated levels of aspartate and asparagine in mutant *KRAS* cells relative to *KRAS* ^+/–^ is consistent with other studies reporting that oncogenic *KRAS* shifts glutamine metabolism toward aspartate synthesis (Ahn & Metallo, 2015; Lyssiotis et al., 2013; Son et al., 2013b). Aspartate is synthesised by transferring the α-amino group on glutamate to the α-keto group of oxaloacetate. This metabolic alteration is thought to have an important role in regeneration of NADPH and NAD to maintaining redox balance in addition to glycolysis (Son et al., 2013a). Additionally, aspartate and glutamine are the precursors for asparagine. Asparagine is involved in the regulation of the cellular adaptation to depletion of glutamine and other nonessential amino acids, as well as suppression of apoptosis induced by glutamine depletion (Zhang et al., 2014).

Glutathione (GSH) levels were also increased in mutant *KRAS* cells compared to their WT, isogenic counterpart (*KRAS* ^+/–^). A higher level of glutathione was also evident in *KRAS* ^G13D/–^ relative to *KRAS* ^G13D/+^ cells. In agreement with our findings, a recent study (Kerr et al., 2016) found that *KRAS* mutation and copy number gain is associated with up-regulation of glutathione metabolism. Glutathione is a tripeptide (consisting of glutamate, cysteine and glycine) that is involved in cellular protection against reactive oxygen species (ROS) and reactive nitrogen (RNS) species (Aquilano et al., 2014)_._ GSH deregulation has been reported in many human cancers (Calvert et al., 1998; Estrela et al., 2006; Obrador et al., 2002), possibly because GSH is protective against oxidative damage (Ortega et al., 2010), stress-induced apoptosis (Franco et al., 2009) and multidrug and radiation resistance (Arrick & Nathan, 1984; Estrela et al., 1995; Meister, 1991; Mitchell & Russo, 1987).

*KRAS* ^G13D/+^ and *KRAS* ^G13D/–^ cells also exhibited higher concentrations of proline relative to *KRAS* ^+/–^ cells. Proline is an α-amino acid that is derived from glutamate via the intermediate pyrroline-5-carboxylate (P5C). P5C is subsequently converted to proline by the NAD (P) H-dependent enzyme pyrroline-5-carboxylate reductase (PYCR), which is present in three isoforms: PYCR1, PYCR2 and PYCRL. Up-regulation of mitochondrial PYCR1 and PYCR2 is observed in a number of cancer types, such as prostate cancer and lymphoma (Ahn & Metallo, 2015; De Ingeniis et al., 2012; Ernst et al., 2002). In addition to biosynthesis, proline catabolism is reportedly down-regulated in a number of tumour types. The first step of proline catabolism is catalysed by proline oxidase, which is proposed as a novel mitochondrial tumour suppressor in human cancers (Liu et al., 2012). In their paper, Liu and colleagues propose proline metabolism as a crucial link in the reprogramming of glutamine and glucose metabolism during tumorigenesis. Therefore, the enzymes involved in proline biosynthesis could provide novel targets for cancer therapy.

In our study, we have observed metabolic variation not only between *KRAS* mutant (*KRAS* ^G13D/+^, *KRAS* ^G13D/–^) cells and wild-type (*KRAS* ^+/–^) cells, but also between *KRAS* ^G13D/+^ and *KRAS* ^G13D/–^ cells. Interestingly, *KRAS* ^G13D/–^ showed higher levels of succinate relative to both *KRAS* ^+/–^ and *KRAS* ^G13D/+^. Accumulation of succinate is associated with the inhibition of prolyl hydroxylases. One of the consequences of this inhibition is the stabilization of the transcription factor hypoxia-inducible factor 1-alpha (HIF-1α) and the generation of a pseudo-hypoxic response reducing oxidative phosphorylation and promoting glycolysis (Selak et al. 2005; Tavares et al., 2015). Stabilisation of HIF1α results in the increased expression of lactate dehydrogenase A (LDHA), which increases the conversion of pyruvate to lactate, and the expression of pyruvate dehydrogenase kinase 1 (PDK1), which inhibits pyruvate dehydrogenase (PDH) and decreases mitochondrial oxidation of pyruvate to acetyl coenzyme A (acetyl-CoA) (Kim et al., 2006; Schulze & Harris, 2012; Zhao et al., 2014). One hypothesis is that under metabolic stress (hypoxia or nutrient deprivation), acetyl-CoA production can be compensated by acetyl-CoA synthase 2 (ACCS2), which converts acetate to acetyl-CoA using ATP to drive the net reaction (Kamphorst et al., 2014; Schug et al., 2015). Our results showing increased levels of succinate, lactate and AMP and decreased concentrations of acetate in *KRAS* ^G13D/–^ cells relative to *KRAS* ^+/–^ and *KRAS* ^G13D/+^ cells support these observations and indicate that the KRAS G13D mutation could impact HIF1α function in the absence of the WT KRAS allele.

In addition, as HIF-1α can also actively repress mitochondrial aerobic metabolism through PDK1 (Papandreou et al., 2006), interruption of the TCA cycle could explain lower levels of ATP in *KRAS* ^G13D/−^ cells compared with *KRAS* ^G13D/+^ and *KRAS* ^±^ cells. Interestingly, we also observed increased levels of creatine and depletion of phosphocreatine in *KRAS* ^G13D/−^ cells relative to *KRAS* ^G13D/+^ and *KRAS* ^±^ cells. This could indicate that in low ATP states, phosphocreatine can be used as a rapidly mobilisable reserve of high-energy phosphates to sustain the energetic requirements of cancer cells.

## Conclusion

Overall, our data provide further insights into nutrient use and the regulation of metabolic pathways driven by oncogenic KRAS. In this study, we have used a robust isogenic system to study the role of a single point mutation in one *KRAS* allele in an isogenic genetic background, hence avoiding confounding genetic factors.

We have demonstrated that the *KRAS*^*G13D*^ oncogene is associated with general metabolic reprogramming to support growth and counter stress, including alterations in the metabolism of amino acids and enhanced glutathione biosynthesis. Additionally, we show that *KRAS*^*G13D/*+^ and *KRAS*^*G13D/−*^ cells have distinct metabolic profiles characterized by dysregulation of and changes in the TCA cycle, the glycolysis and glutathione metabolism pathways, as well as in glutamine, acetate and phosphocreatine. The metabolic adaptation driven by the loss of the *KRAS* WT allele that we have initially described in this paper could contribute to differential responses to anticancer treatments and/or reveal pathways that might be exploited for therapeutic intervention.

## Supplementary Information

Below is the link to the electronic supplementary material.

Supplementary file1 (DOCX 637 kb)

## Data Availability

Original NMR data will be deposited in MetaboLights, EBI, Cambridge UK following publication and can be accessed via the code MTBLS3783 at the following url: https://www.ebi.ac.uk/metabolights/.
