# Supplementary material for: Metabonomics study of the effects of single copy mutant KRAS in the presence or absence of WT allele using human HCT116 isogenic cell lines
Source: Metabolomics. 2021 Nov 25;17(12):104. doi: 10.1007/s11306-021-01852-w (PMC8616861; doi:10.1007/s11306-021-01852-w)
Supplement: Supplementary file 1 — Supplementary file1 (DOCX 637 kb) [file 11306_2021_1852_MOESM1_ESM.docx]

**Supplementary Information: Metabonomics approaches for studying the effects of single copy mutant KRAS in the presence or absence of WT allele using human HCT116 isogenic cell lines**

Authors: Dorna Varshavi^a^, Dorsa Varshavi^a^, Nicola McCarthy^b^, Kirill Veselkov^c^, Hector C Keun^d^ and Jeremy R. Everett^a^*

^a^ Medway Metabonomics Research Group,

University of Greenwich,

Chatham Maritime,

Kent,

ME4 4TB

United Kingdom

^b^ Horizon Discovery Ltd.,

Cambridge Research Park,

8100 Beach Dr, Waterbeach,

Cambridge,

CB25 9TL

United Kingdom

^c^ Department of Surgery and Cancer,

Faculty of Medicine,

Imperial College,

London,

SW7 2AZ

United Kingdom

^d^ Department of Surgery and Cancer,

Imperial College London,

Hammersmith Hospital Campus,

London,

W12 ONN UK

United Kingdom

^e^ Current Address:

Department of Biological Sciences

116 Street & 85 Ave

Edmonton Alberta T6G 2R3

Canada

^f^ Current Address:

Milner Therapeutics Institute

Jeffrey Cheah Biomedical Centre

University of Cambridge

Puddicombe Way

Cambridge CB2 0AW

United Kingdom

* Author for Correspondence

Telephone: +44 (0)208 331 8323

Email: [j.r.everett@greenwich.ac.uk](mailto:j.r.everett@greenwich.ac.uk)

**Supplementary Figure 1.** a) MMC cross validated score plot of the 600 MHz ^1^H NMR spectra of extracts of HCT116 cells, b) superimposed NMR spectra of HCT116 cells, c) a ‘heat map display of the 600 MHz ^1^H NMR spectra of *KRAS* ^G13D/-^ (top 5 strips), *KRAS* ^G13D/+^(middle 5 strips) and *KRAS* ^+/-^ (bottom 5 strips). Red and blue elements in the spectra indicate NMR signals that are more intense, or less intense, respectively, than the median signal intensity for all the samples.

**Supplementary Figure 2.** Leave one out cross validation of the 600 MHz ^1^H NMR spectra of extracts of HCT116 cells using the quadratic as a classifier; The upper left and upper right diagnostic plots indicate the changes in the percentage of correct classification and the variation related to discrimination as a function of the number of discriminating components respectively. The red colour-bar highlights the optimum number of discriminating components. The diagonal of each confusion matrix shows the percentage of samples that are correctly predicted in each class by colouring each box in confusion matrices, black to white with a descending predictivity. The off-diagonal boxes represent the percentage of samples in one class being incorrectly predicted into other classes.


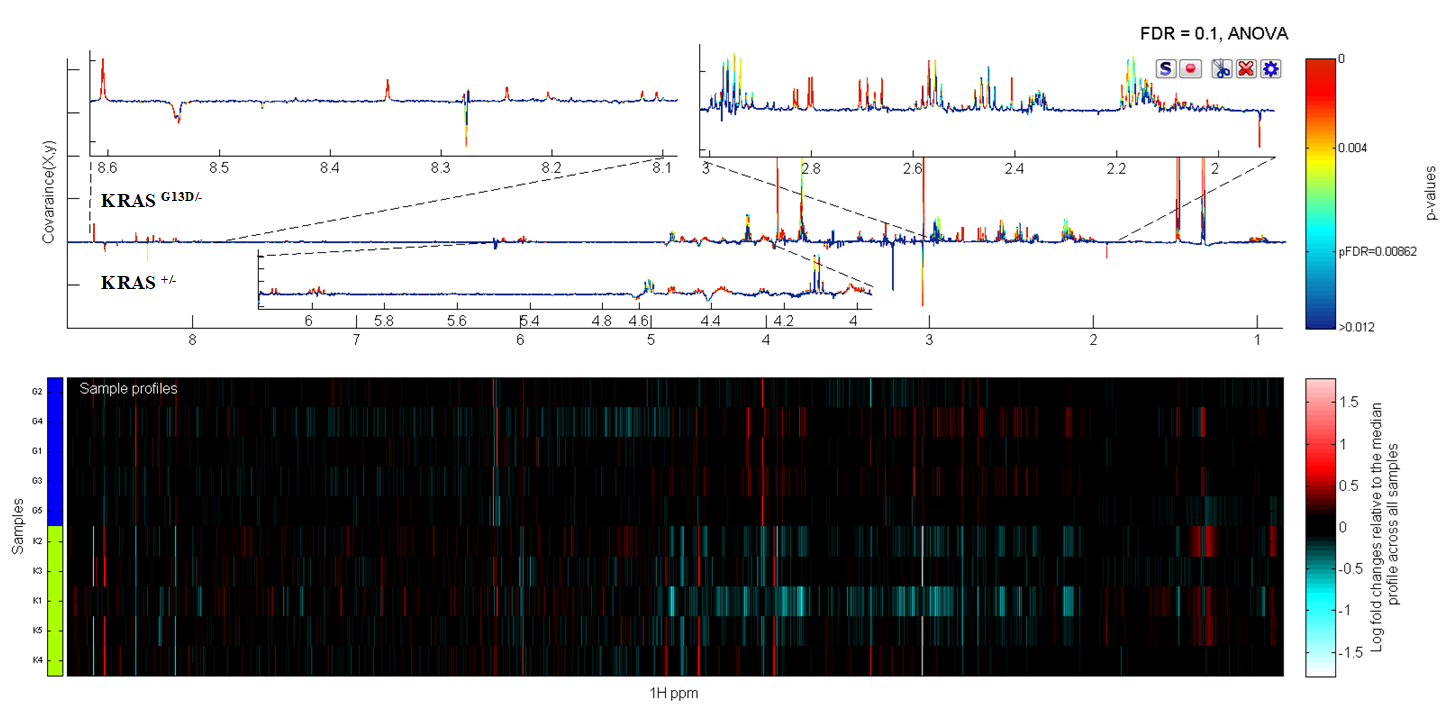


**Supplementary Figure 3.** At bottom a ‘heat map display of the 600 MHz ^1^H NMR spectra of *KRAS* ^G13D/-^ (top 5 strips) vs the corresponding spectra of *KRAS*^+/-^ (bottom 5 strips). Red and blue elements in the spectra indicate NMR signals that are more intense, or less intense, respectively, than the median signal intensity for all the samples. At top, the corresponding ANOVA plot, showing positive peaks for those metabolite signals that are more intense in *KRAS* ^G13D/-^, and negative peaks for those metabolite signals that are less intense. The signals are colour coded by the p value adjusted for FDR of 0.1.


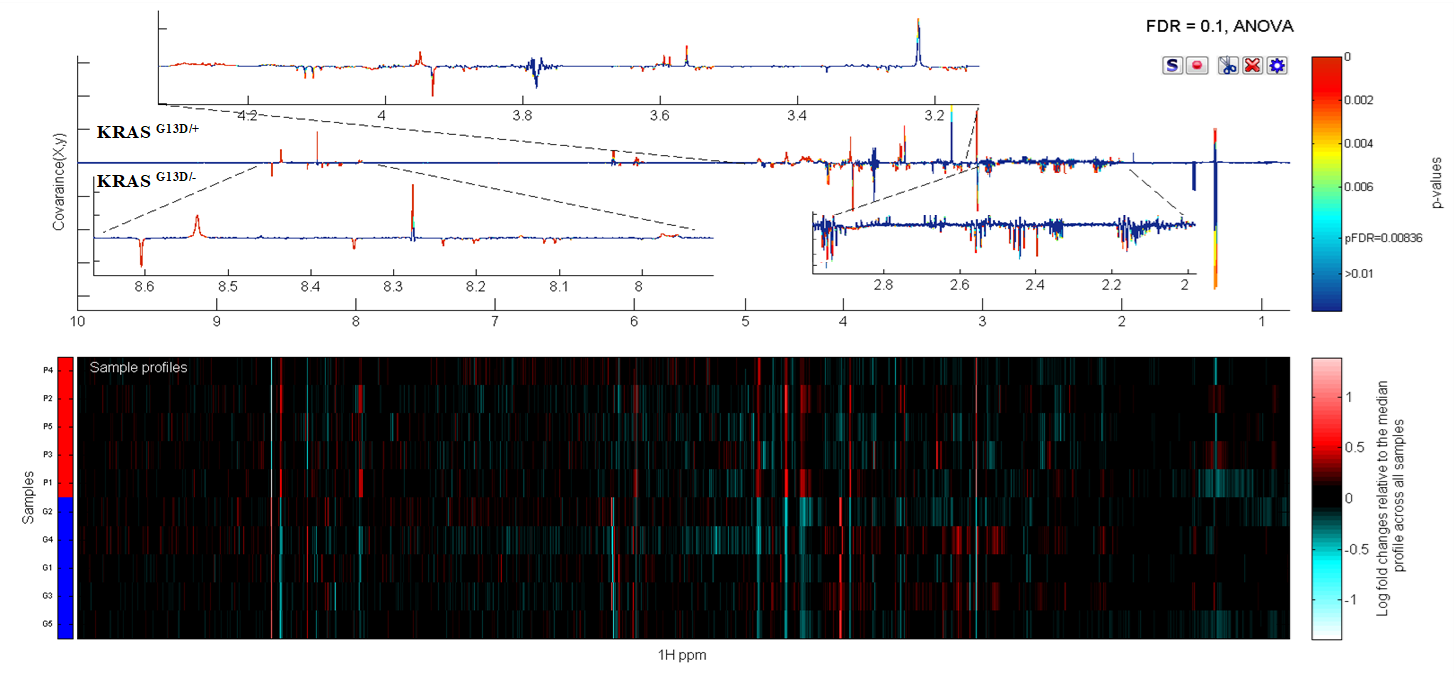
**Supplementary Figure 4.** At bottom a ‘heat map display of the 600 MHz ^1^H NMR spectra of *KRAS* ^G13D/+^ (top 5 strips) vs the corresponding spectra of *KRAS*^G13D/-^ (bottom 5 strips). Red and blue elements in the spectra indicate NMR signals that are more intense, or less intense, respectively, than the median signal intensity for all the samples. At top, the corresponding ANOVA plot, showing positive peaks for those metabolite signals that are more intense in *KRAS* ^G13D/+^, and negative peaks for those metabolite signals that are less intense. The signals are colour coded by the p value adjusted for a false discovery rate (FDR) of 0.1, such that the FDR-adjusted p value for statistical significance is 0.00836. Signals to the ‘red’ side of light blue are statistically significantly discriminating

**Supplementary Table 1.** Data acquisition and processing parameters for 2D NMR spectra of HCT116 *KRAS* ^G13D/-^

| **Parameter** | **JRES** | **COSY** | **HSQC** | **HMBC** |
| --- | --- | --- | --- | --- |
| F2 spectral width in Hz | 10,026.7 | 9,578 | 9,578 | 9,578 |
| F1 spectral width in Hz | 78.0 | 9,578 | 34,729 | 34,729 |
| data points in t2 | 8,192 | 8,192 | 4,096 | 4,096 |
| spectral size in F2 | 8,192 | 8,192 | 4,096 | 4,096 |
| increments in t1 | 40 | 512 | 800 | 400 |
| spectral size in F1 | 512 | 2,048 | 2,048 | 2,048 |
| number of scans | 2 | 80 | 128 | 384 |
| relaxation delay in seconds | 2.00 | 2.00 | 2.00 | 2.00 |
| apodisation | sine bell in t1 and t2 with first point correction in t1 | sine bell squared in t2, sine square with first point correction in t1 | sine bell squared in t2, sine square with first point correction in t1 | sine bell in t1 and t2 with first point correction in t1 |
| Bruker pulse sequence | jresgpprqf | Cosygpprqf | hsqcetgpprsisp 2.2.be | hmbcgplpndprqf |
| notes | the spectrum was tilted by 45^0^ and symmetrised | t1 noise reduction was applied to the spectrum |  |  |

**Supplementary Table 2.** Complete Assigned NMR Spectroscopic Data for the Metabolites Identified in Cell Extracts of Human HCT116 and SW48 Colorectal Cancer Cells (Varshavi *et al.*, 2020)

| **metabolite observed** | **HMDB code^3^** | **HSQC H in ppm** | **HSQC C in ppm** | **HMDB H data in ppm^3^** | **HMDB C data in ppm^3^** | **HMBC 1 in ppm** | **HMBC 2 in ppm** | **HMBC 3 in ppm** | **COSY 1 in ppm** | **COSY 2 in ppm** | **COSY 3 in ppm** | **multiplicity** | **J 1 in Hz** |
| --- | --- | --- | --- | --- | --- | --- | --- | --- | --- | --- | --- | --- | --- |
| pantothenic acid | 00210 | 0.899 | 21.9 | 0.88 | 21.8 | - | - | - | - | - | - | s |  |
| pantothenic acid | 00210 | 0.934 | 32.4 | 0.92 | 23.2 | - | - | - | - | - | - | s |  |
| isoleucine | 00172 | 0.943 | 13.9 | 0.925 | 13.9 | 27.3 | 38.8 | - | 1.274 | 1.462 | - | t | 7.5 |
| leucine | 00687 | 0.961 | 23.8 | 0.94 | 23.6 | 24.8 | 27.0 | 42.7 | 1.723 | - | - | d | 6.4 |
| leucine | 00687 | 0.972 | 24.9 | 0.96 | 24.8 | 23.8 | 27.0 | 42.7 | 1.723 | - | - | d | 6.5 |
| valine | 00883 | 0.996 | 19.5 | 0.97 | 19.4 | 20.8 | 32.0 | 63.2 | 2.279 | - | - | d | 6.9 |
| isoleucine | 00172 | 1.015 | 17.5 | 0.995 | 17.4 | 27.3 | 38.7 | 62.4 | 1.987 | - | - | d | 7.1 |
| valine | 00883 | 1.046 | 20.8 | 1.03 | 20.8 | 19.5 | 32.0 | 63.2 | 2.280 | - | - | d | 7.0 |
| L-lactic acid | 00190 | 1.331 | 22.9 | 1.31 | 22.9 | 71.3 | 185.4 | - | 4.113 | - | - | d | 6.9 |
| threonine | 00167 | 1.335 | 22.3 | 1.316 | 22.3 | 63.3 | 68.8 | - | 4.258 | - | - | d | 6.6 |
| alanine | 00161 | 1.485 | 19.0 | - | - | 53.4 | 178.7 | - | 3.788 | - | - | d | 7.2 |
| leucine | 00687 | 1.691 | 42.7 | - | 42.6 | - | - | - |  | - | - | - | - |
| leucine | 00687 | 1.720 | 27.0 | 1.7 | 26.8 | - | - | - | 0.961 | 3.740 | - | m | - |
| leucine | 00687 | 1.748 | 42.7 | - | 42.6 | - | - | - | - | - | - | - | - |
| acetate | 00042 | 1.919 | 26.2 | 1.9 | 26.1 | 184.3 | - | - | - | - | - | s | - |
| proline | 00019 | 2.011 | 26.6 | - | - | - | - | - | 2.359 | 2.060 | 3.353 | m | - |
| *N*-acetylglutamic acid |  | 2.022 | 24.8 | - | - | - | - | - | - | - | - | - | - |
| pyroglutamic acid | 00267 | 2.034 | 28.2 | 2.010 | 28.0 | - | - | - | 4.180 | 2.510 | 2.407 | - | - |
| proline | 00019 | 2.073 | 31.8 | 2.06 | 31.8 | - | - | - | 4.138 |  |  | m | - |
| glutamate | 00148 | 2.059 | 29.8 | 2.04 | 29.8 | 184.1 | 177.6 | - | 3.762 | 2.356 | 2.114 | m, 2nd order | - |
| UDP-*N-*acetylglucosamine | 00290 | 2.084 | 25.0 | 2.069 | 24.9 | - | - | - | - | - | - | s | - |
| UDP-*N*-acetylgalactosamine | 00304 | 2.088 | 25.0 | 2.08 | 25.0 | - | - | - | - | - | - | s | - |
| glutamate | 00148 | 2.140 | 29.8 | 2.12 | 29.8 | 184.1 | 177.3 | 57.3 | 3.763 | 2.356 | 2.050 | m | - |
| glutamine | 00641 | 2.145 | 29.1 | 2.125 | 29.3 | 57.3 |  |  | 3.760 | 2.460 |  | m, 2nd order | - |
| glutathione | 00125 | 2.171 | 29.1 | 2.158 | 28.9 | 177.7 | 177.0 | 57.0 | 2.560 | 3.785 |  | m, 2nd order | - |
| glutamate | 00148 | 2.355 | 36.4 | 2.34 | 36.4 | 184.1 | 57.5 | 29.8 | 2.100 | - | - | m, 2nd order | - |
| proline | 00019 | 2.359 | 31.9 | - | - | - | - | - | 4.138 | - | - | m | - |
| pyroglutamic acid | 00267 | 2.405 | 32.5 | 2.385 | 32.3 | 61.2 | 28.2 | - | 2.047 | 2.509 | - | - | - |
| succinic acid | 00254 | 2.406 | 37.1 | - | - | 184.6 | - | - |  | - | - | s | - |
| glutamine | 00641 | 2.460 | 33.7 | 2.449 | 33.9 | 180.5 | 56.9 | 29.1 | 2.145 | - | - | m, 2nd order | - |
| **metabolite observed** | **HMDB code^3^** | **HSQC H in ppm** | **HSQC C in ppm** | **HMDB H data in ppm^3^** | **HMDB C data in ppm^3^** | **HMBC 1 in ppm** | **HMBC 2 in ppm** | **HMBC 3 in ppm** | **COSY 1 in ppm** | **COSY 2 in ppm** | **COSY 3 in ppm** | **multiplicity** | **J 1 in Hz** |
| pyroglutamic acid | 00267 | 2.507 | 28.2 | 2.488 | 28.0 | - | - | - | 4.180 | 2.405 | 2.032 | - | - |
| citrate | 00094 | 2.543 | 48.5 | 2.53 | 48.7 | - | - | - | 2.663 | - | - | d | 15.6 |
| glutathione | 00125 | 2.560 | 34.2 | 2.548 | 34.0 | 177.8 | 29.0 |  | 2.173 | - | - | - |  |
| beta-alanine | 00056 | 2.559 | - | 2.53 | 36.4 | - | - | - | 3.184 | - | - | t | 6.7 |
| citrate | 00094 | 2.667 | 48.5 | 2.65 | 48.7 | - | - | - | 2.543 | - | - | d | 15.6 |
| aspartate | 00191 | 2.684 | 39.4 | 2.71 | 39.3 | 55.1 | 180.5 | - | 2.817 | 3.902 | - | dd | 17.4 |
| aspartate | 00191 | 2.816 | 39.4 | 2.8 | 39.5 | 55.0 | 177.1 | 180.4 | 2.687 | 3.902 | - | dd | 17.5 |
| asparagine | 00168 | 2.868 | 37.4 | 2.84 | 37.4 | 54.2 | - | - | 2.957 | 4.008 | - | dd | 7.4 |
| asparagine | 00168 | 2.954 | 37.5 | 2.94 | 37.4 | 54.2 |  | - | 2.860 | 4.008 | - | dd | 4.2 |
| glutathione | 00125 | 2.935 | 28.4 | 2.943 | 28.3 | 58.6 | 174.6 | - | 2.983 | 4.573 | - | dd | 7.2 |
| glutathione | 00125 | 2.980 | 28.4 | 2.943 | 28.3 | 58.6 | 174.6 | - | 2.930 | 4.573 | - | dd | 5.1 |
| creatine | 00064 | 3.041 | 39.8 | 3.03 | 39.5 | 56.7 | 160.0 | - | - | - | - | s | - |
| L-cystine | 00192 | 3.132 | - | 3.18 | 40.6 | - | - | - | 4.092 | 3.392 | - | dd | 15.2 |
| histidine | 00177 | 3.136 | - | 3.15 | 30.2 | - | - | - | 4.002 | 3.298 | - | dd | 14.2 |
| ethanolamine | 00149 | 3.147 | - | 3.130 | 44.2 | - | - | - | 3.829 | - | - | t | 5.3 |
| beta-alanine | 00056 | 3.184 | - | 3.17 | 39.6 | - | - | - | 2.560 | - | - | t | 6.4 |
| phosphorylcholine | 01565 | 3.224 | 56.9 |  |  | 56.8 | 69.3 | - | - | - | - | t | 0.5 |
| beta-D-glucose | 00122 | 3.251 | 77.0 | 3.24 | 77.0 |  |  | - | 4.652 | 3.495 | - | - | - |
| taurine | 00251 | 3.269 | 50.4 | 3.24 | 50.4 | 38.2 |  | - | - | 3.430 | - | t | 6.6 |
| betaine | 00043 | 3.269 | 56.3 |  |  | 69.1 | 56.2 | - | - | - | - | s | - |
| myo-inositol | 00211 | 3.284 | 77.2 | 3.27 | 77.2 | 75.2 |  | - | 3.623 | - | - | t | 9.4 |
| histidine | 00177 | 3.291 | - | 3.25 | 30.2 | - | - | - | 4.000 | 3.137 | - | dd | 14.3 |
| proline | 00019 | 3.345 | 48.9 | 3.32 | 49.0 | - | - | - | 2.014 | 3.425 | - | ddd | - |
| methanol | 01875 | 3.357 | 51.8 | 3.37 | 51.4 | - | - | - |  |  | - | s | - |
| L-cystine | 00192 | 3.391 |  | 3.38 | 40.5 | - | - | - | 4.094 | 3.132 | - | dd | 14.7 |
| beta-D-glucose | 00122 | 3.408 | 72.5 | 3.39 | 72.3 | - | - | - | 3.494 |  | - | dd | 9.8 |
| alpha-D-glucose | 00122 | 3.418 | 72.5 | 3.39 | 72.3 | - | - | - | 3.835 | 3.716 | - | dd | 9.9 |
| proline | 00019 | 3.426 | 48.9 | 3.41 | 49.0 | - | - | - | 2.014 | 3.343 | - | ddd |  |
| taurine | 00251 | 3.427 | 38.2 | 3.40 | 38.3 | - | - | - |  | 3.269 | - | t | 6.7 |
| beta-D-glucose | 00122 | 3.472 | 78.8 | 3.47 | 78.6 | - | - | - | 3.895 | 3.725 | - | ddd | 9.8 |
| beta-D-glucose | 00122 | 3.498 | 78.6 | 3.47 | 78.6 | - | - | - | 3.251 | 3.410 | - | dd | 9.2 |
| myo-inositol | 00211 | 3.540 | 74.0 | 3.52 | 74.0 | 75.2 | - | - | 4.068 | 3.625 | - | dd | 10.0 |
| alpha-D-glucose | 00122 | 3.542 | 74.3 | 3.52 | 74.2 | - | - | - | 5.239 | 3.718 | - | dd | 9.8 |
| UDP-*N-*acetylglucosamine | 00290 | 3.560 | 72.3 | 3.56 | 72.1 | - | - | - | 3.932 | 3.814 | - | dd | 10.1 |
| **metabolite observed** | **HMDB code^3^** | **HSQC H in ppm** | **HSQC C in ppm** | **HMDB H data in ppm^3^** | **HMDB C data in ppm^3^** | **HMBC 1 in ppm** | **HMBC 2 in ppm** | **HMBC 3 in ppm** | **COSY 1 in ppm** | **COSY 2 in ppm** | **COSY 3 in ppm** | **multiplicity** | **J 1 in Hz** |
| glycine | 00123 | 3.562 | 44.3 | 3.54 | 44.3 | 175.3 |  | - | - | - | - | s |  |
| threonine | 00167 | 3.591 | 63.3 | 3.575 | 63.5 | 68.7 | 175.7 | - | 4.257 | - | - | d | 7.1 |
| phosphorylcholine | 01565 | 3.597 | 69.3 | 3.57 | 68.9 | 56.8 | 60.8 | - | 4.168 | - | - | 2nd order m | - |
| L-valine | 00883 | 3.615 | 63.2 | 3.60 | 63.3 |  |  | - | 2.279 | - | - |  | - |
| myo-inositol | 00211 | 3.626 | 75.3 | 3.61 | 75.1 | 77.1 | 74.0 | - | 3.541 | 3.284 | - | dd | 10.1 |
| isoleucine | 00172 | 3.675 | 62.4 | 3.66 | 62.5 | - | - | - | - | - | - | d | 4.0 |
| alpha-D-glucose | 00122 | 3.719 | 75.6 | 3.7 | 75.6 | - | - | - | - | - | - | dd | 9.4 |
| beta-D-glucose | 00122 | 3.729 | 63.6 | 3.74 | 63.4 | - | - | - | 3.900 | 3.469 | - | dd | 12.4 |
| leucine | 00687 | 3.737 | 56.3 | 3.74 | 56.2 | - | - | - | - | - | - | - | - |
| alpha-D-glucose | 00122 | 3.760 | 63.5 | 3.75 | 63.5 | - | - | - | - | - | - | - | - |
| glutamate | 00148 | 3.761 | 57.5 | 3.748 | 57.6 | 36.4 | 29.8 |  | 2.100 | 2.030 | - | dd | 7.2 |
| glutathione | 00125 | 3.782 | 46.2 | 3.766 | 46.0 |  | 179.1 | 174.6 | - | - | - | - | - |
| glutathione | 00125 | 3.782 | 57.0 | 3.766 | 56.8 | 176.9 | 34.3 | 29.0 | 2.170 | - | - | m | - |
| glutamine | 00641 | 3.783 | 57.0 | 3.766 | 57.2 | 176.9 | 33.4 | 29.0 | 2.150 | - | - | m | - |
| UDP-N-acetylgalactosamine | 00304 | 3.770 | 63.8 | 3.78 | 63.9 | - | - | - | 4.193 | - | - | m | - |
| alanine | 00161 | 3.787 | 53.4 | - | - | 178.7 | 19.0 | - | 1.485 | - | - | q | 7.2 |
| uridine | 00296 | 3.811 | 63.4 | 3.801 | 63.6 | - | - | - | 4.139 | 3.911 | - | dd | 12.8 |
| UDP-*N-*acetylglucosamine | 00290 | 3.815 | 73.7 | 3.81 | 73.5 | - | - | - | 3.559 | 3.990 | - | dd | - |
| UDP-*N-*acetylglucosamine | 00290 | 3.820 | 63.1 | 3.84 | 62.8 | - | - | - | 3.950 | - | - | m |  |
| ethanolamine | 00149 | 3.829 |  | 3.81 | 60.6 | - | - | - | 3.148 | - | - | t | 5.2 |
| alpha-D-glucose | 00122 | 3.836 | 74.3 | 3.82 | 74.1 | - | - | - | 3.419 | - | - | - | - |
| alpha-D-glucose | 00122 | 3.842 | 63.5 | 3.83 | 63.5 | - | - | - |  | - | - | - | - |
| inosine | 00195 | 3.845 | 64.1 | 3.859 | 64.0 | - | - | - | 4.283 | - | - | dd | 12.8 |
| serine | 589 | 3.848 | 59.24 | - | - | - | - | - |  | - | - | m | - |
| UDP-*N*-acetylglucosamine | 290 | 3.87 | 63.1 | 3.84 | 62.8 | - | - | - | 3.950 | - | - | m | - |
| beta-D-glucose | 00122 | 3.900 | 63.6 | 3.9 | 63.4 | - | - | - | 3.469 | 3.729 | - | dd | 12.3 |
| aspartate | 00191 | 3.902 | 55.1 | 3.89 | 55.1 | - | - | - | 2.815 | 2.684 | - | dd | 8.7 |
| uridine | 00296 | 3.911 | - | 3.908 | 63.6 | - | - | - | 4.139 |  | - | - |  |
| inosine | 00195 | 3.915 | 64.1 | 3.933 | 64.0 | - | - | - | 4.283 |  | - | dd | 12.8 |
| UDP-*N-*acetylglucosamine | 00290 | 3.931 | 75.8 | 3.91 | 75.6 | - | - | - | 3.800 | 3.563 | - | m | - |
| creatine | 00064 | 3.933 | 56.7 | 3.92 | 56.4 | 39.8 | 160.0 | 177.5 |  | - | - | s | - |
| serine | 00589 | 3.950 | 63.1 | 3.935 | 63.1 | - | - | - | 3.850 | - | - | dd | 12.2 |
| **metabolite observed** | **HMDB code^3^** | **HSQC H in ppm** | **HSQC C in ppm** | **HMDB H data in ppm^3^** | **HMDB C data in ppm^3^** | **HMBC 1 in ppm** | **HMBC 2 in ppm** | **HMBC 3 in ppm** | **COSY 1 in ppm** | **COSY 2 in ppm** | **COSY 3 in ppm** | **multiplicity** | **J 1 in Hz** |
| UDP-N-acetylgalactosamine | 00304 | 3.977 | 70.5 |  | 70.5 | - | - | - | 4.256 | - | - | m | - |
| UDP-*N-*acetylglucosamine | 00290 | 3.994 | 56.5 | 3.99 | 56.2 | - | - | - | 5.522 | 3.818 | - | m | - |
| serine | 00589 | 3.995 | 63.0 | 3.975 | 63.1 | - | - | - | 3.850 |  | - | dd | 12.3 |
| histidine | 00177 | 4.000 |  | 3.98 | 57.3 | - | - | - | 3.290 | 3.140 | - | m | - |
| asparagine | 00168 | 4.008 | 54.1 | 3.99 | 54.1 | - | - | - | 2.870 | 2.940 | - | m | - |
| adenosine 5'-monophosphate | 00045 | 4.012 | 66.3 | 4.04 | 66.2 | - | - | - | 4.368 | - | - | m | - |
| UDP-N-acetylgalactosamine | 00304 | 4.041 | 71.2 | 4.04 | 71.2 | - | - | - | - | - | - | m | - |
| myo-inositol | 00211 | 4.067 | 75.0 | 4.06 | 74.9 | 75.1 | - | - | 3.541 |  | - | t | 2.9 |
| L-cystine | 00192 | 4.092 |  | 4.10 | 56.1 |  | - | - | 3.393 | 3.132 | - | dd | 8.4 |
| L-lactic acid | 00190 | 4.113 | 71.4 | 4.10 | 71.3 | 23.0 | 185.4 |  | 1.331 |  | - | q | 6.9 |
| proline | 00019 | 4.136 | 64.1 | 4.13 | 64.0 | - | - | - | 2.352 | 2.073 | - | dd | 8.8 |
| uridine | 00296 | 4.137 | 87.1 | 4.121 | 87.1 | - | - | - | 4.236 | 3.910 | 3.820 | m | - |
| pyroglutamic acid | 00267 | 4.171 | 60.9 | 4.163 | 61.0 | - | - | - | 2.507 | 2.044 |  | m | - |
| phosphorylcholine | 01565 | 4.171 | 60.9 | 4.15 | 60.6 | - | - | - | 3.598 | - | - | m | - |
| UDP-N-acetylgalactosamine | 00304 | 4.192 | 74.9 |  | 74.9 | - | - | - | 3.770 | - | - | m | - |
| UDP-*N-*acetylglucosamine | 00290 | 4.200 | 67.8 | 4.17 | 67.6 | - | - | - |  | - | - | - | - |
| uridine | 00296 | 4.235 | 72.2 | 4.229 | 72.1 | - | - | - | 4.364 | 4.134 |  | m | - |
| Uridine 5'-diphosphate sugars | - | 4.242 | 67.8 | - |  | - | - | - | - | - | - | - | - |
| UDP-*N-*acetylglucosamine | 00290 | 4.243 | 67.8 | 4.24 | 67.6 | - | - | - | - | - | - | m | - |
| UDP-N-acetylgalactosamine | 00304 | 4.255 | 52.6 | - | 52.6 | - | - | - | 3.977 | - | - | m | - |
| threonine | 00167 | 4.257 | 68.8 | - | - | - | - | - | 1.335 | 3.591 |  | m | - |
| inosine | 00195 | 4.282 | 88.4 | 4.285 | 88.2 | - | - | - | 4.445 | 3.910 | 3.850 | m | - |
| UDP-*N-*acetylglucosamine | 00290 | 4.294 | 86.0 | 4.29 | 85.7 | - | - | - | - | - | - | - | - |
| Uridine 5'-diphosphate sugars (MIX) |  | 4.295 | 86.1 | - | - | - | - | - | - | - | - | - | - |
| uridine | 00296 | 4.360 | 76.4 | 4.344 | 76.5 | - | - | - | 5.922 | 4.236 | - | dd | ca 9.6 Hz |
| **metabolite observed** | **HMDB code^3^** | **HSQC H in ppm** | **HSQC C in ppm** | **HMDB H data in ppm^3^** | **HMDB C data in ppm^3^** | **HMBC 1 in ppm** | **HMBC 2 in ppm** | **HMBC 3 in ppm** | **COSY 1 in ppm** | **COSY 2 in ppm** | **COSY 3 in ppm** | **multiplicity** | **J 1 in Hz** |
| UDP-*N-*acetylglucosamine | 00290 | 4.367 | 72.5 | 4.35 | 72.3 | - | - | - | - | - | - | m | - |
| adenosine 5'-monophosphate | 00045 | 4.374 | 87.6 | 4.37 | 87.1 | - | - | - | - | - | - | m | - |
| UDP-*N-*acetylglucosamine | 00290 | 4.377 | 76.6 | 4.37 | 76.4 | - | - | - | - | - | - | m | - |
| Uridine 5'-diphosphate sugars (MIX) |  | 4.381 | 76.6 |  |  | - | - | - | 5.991 | - | - | - | - |
| inosine | 00195 | 4.442 | 73.2 | 4.42 | 73.1 | - | - | - | 4.284 | 4.780 | - | dd | 5.2 |
| adenosine 5'-monophosphate | 00045 | 4.512 | 73.4 | 4.51 | 73.3 | - | - | - | 4.370 |  | - | dd | 5.1 |
| glutathione | 00125 | 4.572 | 58.6 | 4.557 | 58.4 | 28.2 | 174.5 | 177.8 | 2.980 | 2.930 | - | dd | 6.9 |
| adenosine 5’-triphosphate | 00538 | 4.614 |  | 4.645 | 73.0 | - | - | - | 4.390 |  | - | dd | 5.0 |
| beta-D-glucose | 00122 | 4.652 | 98.8 | 4.63 | 98.7 | - | - | - | 3.252 |  | - | d | 8.0 |
| inosine | 00195 | 4.787 | 76.8 | 4.768 | 76.8 | - | - | - | 6.106 | 4.444 | - | - | - |
| adenosine 5’-triphosphate | 00538 | 4.810 | - | 4.818 | 77.0 | - | - | - | 6.153 | 4.613 | - | m | - |
| glucose | 00122 | 5.239 | 95.0 | 5.22 | 94.9 | - | - | - | 3.542 | - | - | d | 3.8 |
| UDP-*N-*acetylglucosamine | 00290 | 5.523 | 97.3 | 5.508 | 97.0 | 76.3 | - | - | 3.996 | - | - | dd | 7.3 |
| UDP-*N-*acetylgalactosamine | 00304 | 5.556 | 97.5 | 5.54 | 97.5 | - | - | - | 4.255 | - | - | dd | 7.3 |
| UDP-glucose | 00286 | 5.610 | 98.5 | 5.60 | 98.2 | - | - | - | 3.550 | - | - | - | - |
| UDP-galactose | 00302 | 5.648 | - | 5.63 | 98.6 |  | - | - | 3.828 | - | - | - |  |
| uridine | 00296 | 5.907 | 105.1 | 5.890 | 105.1 |  | - | - | 7.877 | - | - | d | 8.0 |
| uridine | 00296 | 5.922 | 92.2 | 5.902 | 92.1 |  | - | - | 4.360 | - | - | d | 4.5 |
| UDP-*N-*acetylglucosamine | 00290 | 5.975 | 105.5 | 5.97 | 105.3 | 144.7 | - | - | 7.955 | - | - | d | 8.2 |
| UDP-*N-*acetylgalactosamine | 00304 | 5.975 | 105.5 | 5.95 | 105.5 | 144.7 | - | - | - | - | - | - | - |
| UDP-*N-*acetylglucosamine | 00290 | 5.990 | 91.3 | 5.97 | 91.0 |  | - | - | 4.381 | - | - | d | - |
| UDP-*N-*acetylgalactosamine | 00304 | 5.990 | 91.3 | 5.97 | 91.4 | - | - | - | - | - | - | - | - |
| NAD | - | 6.047 | 89.5 | 6.01 | 89.3 | - | - | - |  | - | - | d | 5.6 |
| inosine | 00195 | 6.107 | 91.2 | 6.055 | 91.0 | 76.8 |  |  | 4.777 | - | - | d | 5.7 |
| NAD | - | 6.101 | 102.9 | 6.08 | 102.6 | - | - | - |  | - | - |  |  |
| adenosine 5'-monophosphate | 0000045 | 6.147 | 89.6 | 6.1 | 89.6 | - | - | - |  | - | - | d | 5.9 |
| adenosine 5’-triphosphate | 00538 | 6.153 | - | 6.148 | 89.3 | - | - | - | 4.811 | - | - | d | 5.5 |
| **metabolite observed** | **HMDB code^3^** | **HSQC H in ppm** | **HSQC C in ppm** | **HMDB H data in ppm^3^** | **HMDB C data in ppm^3^** | **HMBC 1 in ppm** | **HMBC 2 in ppm** | **HMBC 3 in ppm** | **COSY 1 in ppm** | **COSY 2 in ppm** | **COSY 3 in ppm** | **multiplicity** | **J 1 in Hz** |
| tyrosine | - | 6.907 | 118.7 |  |  | - | - | - | 7.200 | - | - | d | 8.5 |
| histidine | 00177 | 7.100 |  | 7.09 | 120.0 | - | - | - |  | - | - | s v. broad |  |
| tyrosine | - | 7.200 | 133.6 |  |  | - | - | - | 6.907 | - | - | d | 8.6 |
| phenylalanine | - | 7.342 | 132.2 | 7.36 | 130.4 | - | - | - |  | - | - |  |  |
| phenylalanine | - | 7.435 | 132.1 | 7.41 | 131.8 | - | - | - |  | - | - |  |  |
| uridine | 00296 | 7.877 | 144.7 | 7.86 | 144.6 | - | - | - | 5.907 | - | - | d | 8.1 |
| histidine | 00177 | 7.900 |  | 7.90 | 138.4 | - | - | - |  | - | - | s v. broad |  |
| Uridine 5'-diphosphate sugar (s) | - | 7.955 | 144.4 | - | - | - | - | - | 5.980 | - | - | d | 8.1 |
| Uridine 5'-diphosphate sugar (s) | - | 7.957 | 144.4 | - | - | - | - | - | 5.980 | - | - | d | 8.1 |
| UDP-*N-*acetylgalactosamine | 00304 | 7.955 | 144.4 | 7.94 | 144.5 | - | - | - | - | - | - | - | 8.1 |
| UDP-*N-*acetylglucosamine | 00290 | 7.958 | 144.4 | 7.94 | 144.3 | 169.4 | 154.9 | - | 5.975 | - | - | d | 8.1 |
| NAD | - | 8.205 | 131.4 | 8.19 | 131.1 | - | - | - | - | - | - | - | - |
| inosine | 00195 | 8.241 | 149.2 | 8.187 | 148.9 | 161.6 | - | - | - | - | - | s | - |
| adenosine 5'-monophosphate (AMP) | 00045 | 8.273 | 155.6 | 8.254 | 155.3 | - | - | - | - | - | - | - | - |
| adenosine 5’-triphosphate (ATP) | 00538 | 8.277 | 155.7 | 8.257 | 155.4 | - | - | - | - | - | - | s | - |
| inosine | 00195 | 8.348 | 143.1 | 8.305 | 142.7 | 151.4 | 127.1 | - | - | - | - | s | - |
| NAD | - | 8.429 | 142.6 | 8.4 | 142.4 | - | - | - | - | - | - |  | - |
| adenosine 5’-triphosphate (ATP) | 00538 | 8.539 | 142.9 | 8.546 | 142.5 | - | - | - | - | - | - | s | - |
| adenosine 5'-monophosphate (AMP) | 0000045 | 8.614 | 143.1 | 8.61 | 142.6 | - | - | - | - | - | - | s | - |
| NAD | - | 9.146 | 145.2 | 9.15 | 145.1 | - | - | - | - | - | - | - | - |
| NAD | - | 9.343 | 142.8 | 9.33 | 142.5 | - | - | - | - | - | - | - | - |

Footnotes:

1. For multiplicities, s = singlet, d = doublet, t = triplet, q = quartet, m = multiplet, dd = doublet of doublets etc
2. For 2D COSY and 2D HMBC experiments, up to three correlated peaks are noted for each proton of each metabolite i.e. COSY 1, COSY 2 and COSY 3
3. For each metabolite, the code number from the Human Metabolite Database (HMDB) is given together with the reported proton and carbon-13 NMR chemical shifts from that database for comparison with the values reported here

**Supplementary Table 3**. Log_2_ fold changes of discriminating metabolites between genotypes KRAS ^G13D/+^ vs KRAS^+/-^, KRAS ^G13D/-^ vs KRAS^+/-^ and KRAS ^G13D/-^ vs KRAS ^G13D/+^

| **Metabolites** | **KRAS^G13D/+^ vs KRAS^+/-^** | | | | **KRAS ^G13D/-^ vs KRAS^+/-^** | | | **KRAS ^G13D/-^ vs KRAS ^G13D/+^** | | |
| --- | --- | --- | --- | --- | --- | --- | --- | --- | --- | --- |
|  | **ppm** | **p-values** | **q-values** | **Log2 FC** | **p-values** | **q-values** | **Log2 FC** | **p-values** | **q-values** | **Log2 FC** |
| isoleucine | 0.944 | 1.10E-03 | 6.16E-02 | 0.316 | 1.16E-03 | 3.35E-02 | 0.504 | - | - | - |
| leucine | 0.966 | 3.02E-03 | 9.52E-02 | 0.506 | 1.02E-03 | 3.13E-02 | 0.565 | - | - | - |
| valine | 1.041 | 2.05E-03 | 8.05E-02 | 0.400 | 1.72E-03 | 4.02E-02 | 0.405 | - | - | - |
| lactate | 1.337 | - | - | - | 8.92E-04 | 2.95E-02 | 0.884 | 2.13E-03 | 4.81E-02 | 0.658 |
| threonine | 1.340 | 5.76E-04 | 4.60E-02 | 1.199 | 5.30E-03 | 7.54E-02 | 0.671 | 3.22E-03 | 6.26E-02 | -0.575 |
| alanine | 1.478 | 1.98E-03 | 7.96E-02 | 0.833 | 3.13E-04 | 1.88E-02 | 0.983 | - | - | - |
| acetate | 1.919 | - | - | - | 4.14E-04 | 1.91E-02 | -0.792 | 6.79E-03 | 8.56E-02 | -0.617 |
| glutamate | 2.351 | 7.23E-04 | 5.09E-02 | 0.424 | 2.13E-03 | 4.49E-02 | 0.525 | - | - | - |
| succinate | 2.406 | - | - | - | 1.50E-04 | 1.40E-02 | 0.934 | 9.01E-06 | 1.31E-03 | 0.845 |
| glutamine | 2.452 | - | - | - | 1.39E-03 | 3.65E-02 | 0.764 | 3.66E-04 | 1.47E-02 | 0.587 |
| glutathione | 2.557 | 1.66E-03 | 7.38E-02 | 0.612 | 4.22E-04 | 2.10E-02 | 0.724 | 1.23E-03 | 3.31E-02 | 0.459 |
| aspartate | 2.805 | 1.34E-05 | 8.99E-03 | 1.173 | 2.32E-05 | 6.79E-03 | 0.946 | - | - | - |
| asparagine | 2.873 | 3.29E-03 | 9.78E-02 | 0.476 | 3.30E-03 | 5.75E-02 | 0.405 | - | - | - |
| creatine | 3.040 | 5.08E-04 | 4.30E-02 | 0.317 | 2.27E-03 | 4.66E-02 | 0.952 | 4.39E-04 | 1.66E-02 | 0.533 |
| creatine phosphate | 3.045 | - | - | - | 1.69E-03 | 4.01E-02 | -1.483 | 5.11E-04 | 1.84E-02 | -1.827 |
| choline | 3.208 | - | - | - | 1.21E-03 | 3.39E-02 | 0.725 | 7.60E-04 | 2.39E-02 | 0.566 |
| phosphocholine | 3.225 | - | - | - | - | - | - | 5.35E-03 | 8.67E-02 | -0.216 |
| taurine | 3.268 | 1.59E-03 | 7.22E-02 | 0.542 | 5.00E-04 | 2.24E-02 | 0.567 | - | - | - |
| betaine | 3.270 | - | - | - | 1.34E-05 | 5.83E-03 | 0.785 | 5.98E-07 | 3.01E-04 | 0.618 |
| glycine | 3.562 | 3.05E-03 | 6.45E-02 | 0.539 | - | - | - | 4.21E-03 | 7.43E-02 | -0.673 |
| serine | 3.982 | 2.26E-03 | 8.43E-02 | 0.602 | 3.95E-04 | 2.07E-02 | 0.433 | 6.03E-03 | 9.31E-02 | -0.285 |
| myoinositol | 4.067 | 3.10E-03 | 9.56E-02 | -0.429 | - | - | - | 5.09E-06 | 8.60E-04 | -0.508 |
| proline | 4.139 | 1.55E-03 | 7.15E-02 | 0.428 | 5.12E-04 | 2.26E-02 | 0.341 | - | - | - |
| ATP | 8.277 | 2.23E-04 | 2.73E-02 | 0.668 | 2.61E-03 | 5.05E-02 | -1.092 | 1.22E-06 | 3.90E-04 | -1.956 |
| Inosine | 8.349 | - | - | - | 1.17E-06 | 2.40E-03 | 2.084 | 6.71E-07 | 2.92E-04 | 2.077 |
| AMP | 8.614 | - | - | - | 4.81E-04 | 2.05E-02 | 1.413 | 2.49E-08 | 3.61E-05 | 1.906 |
